# Evaluation of the Prediction Potential of the HIrisPlex-S System in a North German Population

**DOI:** 10.3390/genes17040452

**Published:** 2026-04-13

**Authors:** Amke Caliebe, Luisa Bruder, Johanna Riege, Maria Seidel

**Affiliations:** 1 Epidemiolgy, Medical Biometry and Medical Informatics, Health and Medical University Erfurt, 99084 Erfurt, Germany; 2 Institute of Medical Statistics, University Hospital Schleswig-Holstein, Kiel University, 24105 Kiel, Germany; 3 Department of Forensic Genetics, Institute of Legal Medicine, Charité Berlin, 13353 Berlin, Germany; 4 Institute of Legal Medicine, University Hospital Schleswig-Holstein, 24105 Kiel, Germany

**Keywords:** FDP, HIrisplex-S, phenotyping, eye color, hair color and skin color, German population, intermediate phenotypes, SNaPshot

## Abstract

Background: Phenotype prediction for eye, hair and skin color is used in a variety of forensic applications, such as trace analysis, the identification of unknown individuals, and analysis of historical DNA traces. The aim of this study was to evaluate the predictive accuracy of the HIrisPlex-S system in a homogeneous North German population. Methods: A cohort of 155 individuals from this population was sampled, and the 41 HIrisPlex-S SNPs were genotyped using the SNaPshot workflow. In addition, the participants assessed their own eye, hair, and skin color using a standardized questionnaire. The statistical analysis included the calculation of diagnostic indicators such as sensitivity (Sens), specificity (Spec), positive and negative predictive values, and accuracy (Acc). In addition, ROC analyses were performed. Results: The results indicated that predictions of skin and hair color were less accurate, whereas eye color could be determined more reliably. Brown and blue eye colors in particular were predicted accurately (brown: Sens = 94.7%, Spec = 87.7%, Acc = 89.5%; blue: Sens = 98.5%, Spec = 57.7%, Acc = 75.7%), while intermediate eye color (Sens = 0.0%, Spec = 100.0%, Acc = 69.1%), hair color and skin color were difficult to differentiate (e.g., blond hair color: Sens = 80.8%, Spec = 56.0%, Acc = 68.2% and pale skin color: Sens = 73.8%, Spec = 44.8%, Acc = 57.2%). Conclusions: In our study, the HIrisPlex-S system primarily provided rough directional information and could distinguish between very different phenotypes but reached its limits when it comes to similar characteristics.

## 1. Introduction

If no match is found for a trace STR profile, forensic DNA phenotyping (FDP) can be helpful in police investigations by narrowing down the number of persons of interest or identifying unknown deceased persons. FDP performs probability-based prediction of externally visible characteristics (EVCs) such as eye, hair and skin color and of externally non-visible characteristics, such as age and biogeographical origin, solely from the biological trace material [1,2].

Over the past decades, genome-wide association studies have identified many trait-informative genes and SNPs (single nucleotide polymorphisms), on the basis of which forensic assays and statistical prediction models have been developed [3]. Among EVCs, a person’s eye, hair and skin color currently yield the most reliable predictions in practice [1,3,4,5]. The HIrisPlex-S system, based on 41 autosomal SNPs, is the most widely adopted method for the simultaneous prediction of these three traits [4,5]. The 41 SNPs comprise 24 from the original HIrisPlex system for eye and hair color prediction plus 17 from the ‘S’ (skin) extension [5,6]. Many forensic laboratories have already established and forensically validated the HIrisPlex-S system for accreditation purposes and offer phenotyping as a service for investigating authorities. In December 2019, extended DNA analyses of trace material to determine external characteristics for the purpose of criminal prosecution were legally permitted in Germany, provided the trace donor is unknown (German Code of Criminal Procedure: § 81e [3]). Legally admissible external characteristics are eye color, hair color, skin color and age.

Nevertheless, studies investigating the applicability and the validity of the predictions of HIrisPlex-S in specific populations are still rare [6,7,8,9] and completely lack the German population. Our study therefore makes an important contribution to filling this gap. Here, we present the first population study on the HIrisPlex-S system in a German cohort (*n* = 155), with a critical perspective on the interpretation of the data obtained and a well-founded biostatistical evaluation.

## 2. Material and Methods

### 2.1. Sample Collection and Questionnaires

The inclusion criteria of the population study were age of at least 18 years (adults), capacity to give informed consent, residence in Berlin or Brandenburg, West European ancestry, ability to comply with study procedures and provision of signed written informed consent. The study was approved by the ethics committee of the Charité Hospital in Berlin (EA4/148/21) and the ethics committee of the Medical Faculty of Kiel University (B 265/23). Two buccal swabs were collected from each participant using cotton swabs (Heinz Herenz Medizinalbedarf, Hamburg, Germany). A questionnaire (Supplementary Figures S1 and S2) was used to record sex and self-assessed eye, hair and skin color (Supplementary Tables S1 and S2).

### 2.2. DNA Extraction

DNA was extracted from the samples using the EZ1 DNA blood 200 µL Kit on a 6 GC Magtration System M6 (Qiagen, Hilden, Germany). The isolated DNA was eluted in 50 μL ultrapure water. DNA extracts were quantified using the QuantiFluor^®^ ONE ds DNA Kit on the Quantus fluorometer (Promega, Walldorf, Germany). Both methods were applied following the manufacturer’s guidelines and the corresponding manuals for the kits (EZ1 DNA Blood Handbook, QuantiFluor® ONE dsDNA System Technical Manual).

### 2.3. Genotyping of HIrisPlex-S Markers

The genotyping of the 41 autosomal SNPs for the determination of eye, hair and skin color is based on the publications of Walsh et al. [6,10] for the 24-plex assay and Chaitanya et al. [5] for the 17-plex assay.

For practical reasons, the length and thus the position in the electrophoresis assay of some single-base extension (SBE) primers were changed compared to the source publications (Supplementary Tables S3 and S4). Most of the changes led to a shortening of the primers in order to obtain a more cost-effective assay. The orientation of the SBE primers was not modified. In the 24-plex panel, the lengths of the markers rs1805005, rs885479, rs1805008, rs12821256, rs4959270, rs12913832, rs2378249, rs12896399, rs1393350, and rs683 were changed, and in the 17-plex panel, the lengths of the markers rs1667394, rs1126809, rs1426654, rs1545397, rs6059655, rs12441727, rs3212355 and rs8051733 were adjusted. All changes related to the length of the non-specific sequence, except for the marker rs6059655. For better binding of the primer to its template, the specific sequence was increased by four nucleotides, but the overall length was not changed.

Primer concentrations of the PCR reactions (24- and 17-plex) and the cycler conditions were adopted unchanged from the original publications. Both PCR amplifications were adapted to the use of the Qiagen Multiplex Mastermix and performed in a total volume of 9 µL, containing 5 µL Qiagen Multiplex Mastermix, PCR primers in specified concentrations and 1 µL genomic DNA (optimal DNA input at 0.5–1 ng) in a Thermocycler DNA Engine Dyad Peltier (Bio-Rad Laboratories, Feldkirchen, Germany). After PCR clean-up with ExoSAP-IT (Thermo Fisher Scientific, Darmstadt, Germany) the single-base extension (SBE) multiplex reactions were performed in a total volume of 5 µL, containing 1 µL ABI Prism^®^ SNaPshot chemistry (Thermo Fisher Scientific), SBE primers in specified concentrations (Supplementary Tables S3 and S4) and 1 µL cleaned-up PCR product. The primer concentrations of the SBE reactions were adjusted according to the performance of the primer and the peak heights in the electropherograms of the test runs. Lastly, SBE products were cleaned up with SAP enzyme and analyzed on a 3500 Genetic Analyzer (Thermo Fisher Scientific) with POP-4 on a 36 cm capillary length array. The run parameters were as follows: injection voltage of 1.2 kV for 15 s and run time of 800 s at 60 °C. Electropherograms were evaluated with the GeneMapper IDX with self-created panels for automated allele calling (there are example electropherograms in Supplementary Figures S3 and S4). For quality control and to identify systematic genotyping errors, in Supplementary Table S5, the minor allele frequencies (MAFs) of the 41 SNPs analyzed in our population study were compared with those of the European subpopulation in the NCBI database (https://www.ncbi.nlm.nih.gov/snp; accessed on 25 September 2025).

### 2.4. Matching of Phenotype Categories of Questionnaire and HIrisPlex-S

To match the data collected in the questionnaire with the categories predicted by HIrisPlex-S, classification mapping was performed for all traits. Self-reported eye colors were grouped into three categories: blue, brown and intermediate. Eye colors that could not be clearly classified as blue or brown, such as green or a mix of multiple colors, were described as intermediate (for details, see Supplementary Table S1). Hair color was classified as blond, brown, black or red. Due to the small sample size, the four individuals with self-reported red hair color were excluded from the hair color analysis. In the questionnaire, the “Fitzpatrick” classification [11] to assess skin color was applied. It is based on six different skin types defined by color, the ability to tan and the susceptibility to sunburn. However, the HIrisPlex-S system has five different skin color categories: very pale, pale, intermediate, dark and dark-to-black. To align the two classification systems, we mapped the Fitzpatrick scale for skin with the corresponding HIrisPlex-S categories, as described in Walsh et al. [4]: Fitzpatrick scale one was mapped to very pale skin color and two to pale skin color in HIrisPlex-S. Fitzpatrick scales three (light brown, which usually tans) and four (moderate brown, which tans well) were merged into the intermediate HIrisPlex-S category. Lastly, Fitzpatrick scale five was mapped to the dark category and six to the dark-to-black HIrisPlex-S category (Supplementary Tables S1 and S2).

### 2.5. Phenotype Prediction with HIrisPlex-S

Electropherogram results were compiled into Excel sheets. To ensure accuracy, an independent reviewer conducted a second verification of the SNP genotypes. These CSV files with the genotypes were uploaded to the HIrisPlex-S platform (https://hirisplex.erasmusmc.nl, accessed on 25 May 2023) according to the procedure described in the HIrisPlex-S webtool manual (Version 2.0 2018, https://hirisplex.erasmusmc.nl/pdf/hirisplex.erasmusmc.nl.pdf, accessed on 25 May 2023). Afterwards, the results were downloaded from the website. For each individual, the results provide the estimated probability for each category of eye, hair, and skin color. As the predicted category for each phenotype, we selected the one with the highest estimated probability provided by HIrisPlex-S for each individual, in accordance with the standard procedure of the tool. An exception is the analyses in Section 3.5, where a probability threshold was applied. The prediction procedure remained as described above, i.e., selecting the category with the highest estimated probability provided by HIrisPlex-S. However, predictions for which this highest probability fell below a specified threshold were discarded. Another approach for hair color is suggested in [10]. We did not apply this approach in our study for two reasons: (i) we did not have a sufficiently detailed classification of hair color (e.g., no distinction between light brown and dark brown), and (ii) the suggested approach did not always yield a unique hair color category.

### 2.6. Statistical Analysis

All statistical analyses were performed with the statistics software R version 4.2.3 (2023-03-15 ucrt) [12].

The prediction accuracy of different trait manifestations was calculated using contingency tables. For each manifestation of eye, hair and skin color, calculations were performed for binary outcomes, such as blond vs. non-blond. The performance metrics derived were sensitivity, specificity, negative and positive predictive values, area under the receiver operating characteristic curve (AUC) and accuracy.

The AUC was calculated using the “pROC package” v1.19.1 [13]. An AUC value of 1 indicates the best prediction, while 0.5 equals random chance. This package was also used to generate receiver operating characteristic (ROC) curves.

Furthermore, boxplots were created to visualize the difference(s) in probabilities generated by HIrisPlex-S for a given trait expression among the subgroups of that trait. For example, we compared the probability of blond hair calculated by HIrisPlex-S in the subgroups of self-reported blond, brown and black hair.

## 3. Results

### 3.1. Eye, Hair and Skin Phenotypes and HIrisPlex-S Genotypes

We recruited 155 volunteers of self-reported West European ancestry from northern Germany. Three individuals were excluded due to twin status and quality issues, leaving a final sample of 152 participants (54 males, 36%; 98 females, 64%). All of them completed a questionnaire on eye, hair and skin color.

Eye color was highly variable: 44% of participants reported blue eyes and 25% brown (Table 1). The frequency of intermediate eye color was relatively high at 31%. Hair color was predominantly blond (48%) or brown (44%), whereas black (5%) and red (3%) hair were rare. No participant reported dark or dark-to-black skin. Instead, most described their complexion as intermediate (54%) or pale (43%), with very pale skin accounting for only 3%.

The analysis of the HIrisPlex-S markers resulted in 41 complete SNP profiles for all participants. Four SNPs (rs312262906, rs1805006, rs201326893, and rs6119471) were monomorphic in our study population. The MAFs from our population study show high concordance with the MAFs of the European subpopulation in the NCBI database (Supplementary Table S5).

To evaluate the prediction performance of HIrisPlex-S, we assigned the category with the highest probability from HIrisPlex-S as the predicted one for all three phenotypes.

### 3.2. Prediction Performance of HIrisplex-S for Eye Color

**Figure 1:**
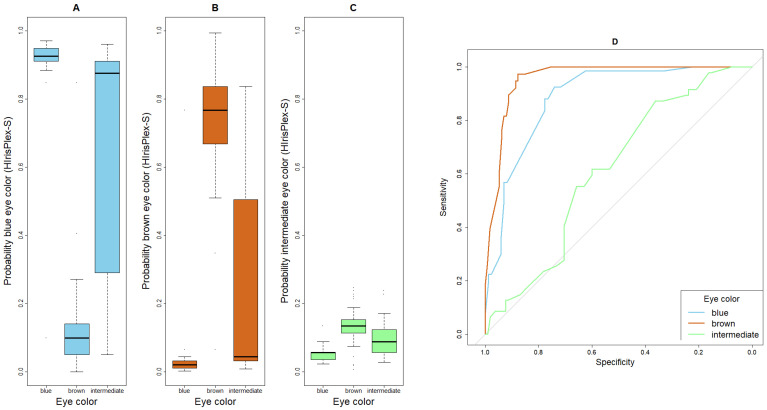
Boxplots and ROC curves for the probability of eye colors, as given by HIrisPlex-S. (**A**) Boxplot for the probability of blue eye color, (**B**) boxplot for the probability of brown eye color, (**C**) boxplot for the probability of intermediate eye color, (**D**) ROC curves of blue, brown and intermediate eye color. The grey 45° diagonal line serves as the ROC curve of random classification.

Of the 67 participants with self-reported blue eye color, 66 were correctly predicted by HIrisPlex-S (Table 2), resulting in a sensitivity of 99% (Table 3). A similarly high sensitivity was observed for brown eyes (36/38 correct, Table 2, sensitivity = 95%, Table 3). Intermediate eye color, on the other hand, was never correctly predicted. For the 47 participants who reported it, 34 were classified as blue and 13 as brown (sensitivity = 0%). Figure 1A shows that the probability of blue eye color, as calculated by HIrisPlex-S, was highest in individuals with blue eyes, as expected, but also elevated in those with intermediate eye color. The probability of brown eye color was clearly the highest in individuals with brown eyes, as desired (Figure 1B). The probability of intermediate eye color was consistently very low across all eye colors, explaining why no individual (with intermediate eye color or other) was classified as having intermediate eye color by HIrisPlex-S (Figure 1C). Specificities were 58% for blue, 88% for brown and 100% for intermediate eye color. The corresponding AUC values were 89%, 96% and 61%, respectively. The ROC curves indicate the best prediction performance for brown eye color, closely followed by blue eye color, whereas the predictions for intermediate eye color were only slightly better than chance (Figure 1D).

### 3.3. Prediction Performance of HIrisplex-S for Hair Color

**Figure 2:**
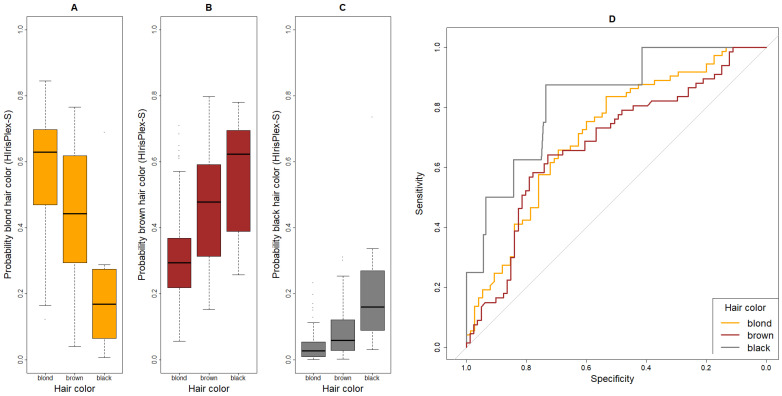
Boxplots and ROC curves for the probability of hair colors, as given by HIrisPlex-S. (**A**) Boxplot for the probability of blond hair color, (**B**) boxplot for the probability of brown hair color, (**C**) boxplot for the probability of black hair color, (**D**) ROC curves of blond, brown and black hair color. The grey 45° diagonal line serves as the ROC curve of random classification.

For hair color, individuals with red hair were excluded due to small sample size (*n* = 4). The highest sensitivity was observed for blond hair: 59 out of 73 individuals were correctly predicted (81%, Table 2 and Table 3). For brown hair, only 35 out of 67 individuals with brown hair were correctly classified (sensitivity of 52%), while 32 were misclassified as blond. Among the eight individuals with black hair, one was correctly predicted by HIrisPlex-S (sensitivity = 13%), whereas six were classified as brown and one as blond. Notably, no participant was incorrectly classified as having black hair. Specificities were 56% for blond, 79% for brown and 100% for black hair. As shown in Figure 2A, the predicted probability for blond hair, as calculated by HIrisPlex-S, was highest for individuals with blond hair color. The highest probability for brown hair color, in contrast, appeared in individuals with black hair (Figure 2B). Finally, the probability for black hair, while highest in individuals with black hair, was generally low, explaining why only one person was classified as having black hair (Figure 2C). The AUC values were in a medium range for blond and brown hair (71% and 68%) and higher for black hair (AUC = 83%) (Table 3, Figure 2D). The latter estimate should be interpreted with caution due to the small sample size (*n* = 8).

### 3.4. Prediction Performance of HIrisPlex-S for Skin Color

**Figure 3:**
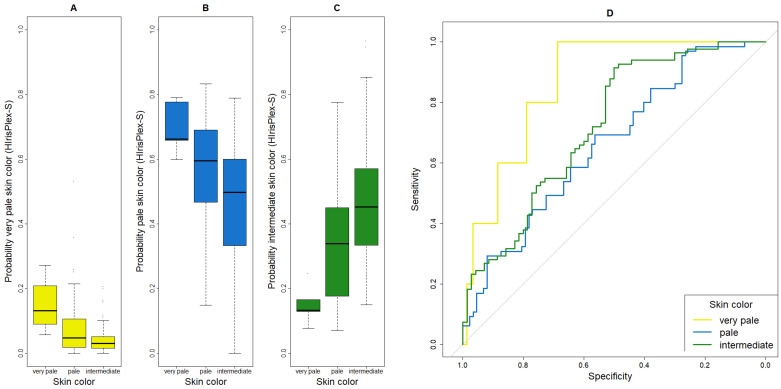
Boxplots and ROC curves for skin color, as given by HIrisPlex-S. (**A**) Boxplot for the probability of very pale skin color, (**B**) boxplot for the probability of pale skin color, (**C**) boxplot for the probability of intermediate skin color, (**D**) ROC curves of very pale, pale and intermediate skin color. The grey 45° diagonal line serves as the ROC curve of random classification.

For skin color, all five individuals with self-reported very pale skin were classified as pale by HIrisPlex-S (sensitivity = 0%, Table 2 and Table 3). Most participants with pale skin were predicted correctly (48/65, 74%). Of the 82 individuals with intermediate skin color, 43 were predicted as pale, 37 as intermediate and two as dark (sensitivity = 45%). The specificities were 99.32% for very pale, 45% for pale and 77% for intermediate skin. The AUC values were 86% (very pale), 66% (pale) and 72% (intermediate). The HIrisPlex-S probabilities for very pale skin color were overall very low, with the highest probabilities for very pale skin (Figure 3A). For the probabilities for pale skin color, individuals with very pale skin color showed the highest values, which explains why these five people were classified as pale (Figure 3B). Individuals with intermediate skin showed the highest values for the probability of intermediate skin color (Figure 3C). Very pale skin color presented the best ROC curve (Figure 3D).

### 3.5. Probability Thresholds for Eye and Hair Color

For eye and hair color, it is sometimes proposed to only accept predictions if the predicted probability of HIrisPlex-S for a given eye or hair color category is above 0.70 [5]. Accordingly, the category with the highest estimated probability was selected as the predicted outcome (as before), and predictions with a maximum probability below 0.70 were excluded. The rationale is to ensure higher prediction reliability by omitting uncertain classifications. We therefore repeated our analyses applying this threshold.

For eye color, 136 out of 152 individuals had a predicted probability above 0.70 for the most likely eye color category (Supplementary Table S6A). A comparison of the performance metrics (AUC, sensitivity, specificity, and predictive values) revealed only minor changes, indicating no gain in prediction performance when applying the probability thresholds (Supplementary Table S7). For hair color, only 38 of 148 individuals (26%) exceeded the 0.70 probability threshold (Supplementary Table S6B). For blond hair, the performance increased with sensitivity and AUC, rising from 81% and 71% to 90% and 79%, respectively (Supplementary Table S7). The specificity dropped from 56% to 45%. Black hair predictions also improved: sensitivity and AUC increased from 13% and 83% to 33% and 96%, while specificity remained at 100%. It is important to note that only three individuals with black hair remained in the reduced dataset. For brown hair, the AUC and sensitivity decreased, while specificity improved. The boxplots and ROC curves related to these analyses are presented in Supplementary Figures S5 and S6. Because only predictions with high probabilities were retained, the distribution of probabilities in the reduced dataset, as displayed in the boxplots, showed extremer values with lower and higher probabilities.

## 4. Discussion

### 4.1. Eye Color

The HIrisPlex-S system demonstrated varying levels of predictive accuracy in our German population sample. Among the three traits, eye color showed the highest prediction accuracy, with 76% for blue, 69% for intermediate and 89% for brown eyes. While individuals with brown and blue eyes are generally well-classified, intermediate colors, such as green and mixtures of different colors, are frequently misclassified. All individuals with intermediate eye colors (*n* = 47) were misclassified as blue (*n* = 34) or brown (*n* = 13). The problem of identifying intermediate eye color was already apparent in the initial Irisplex and HIrisPlex studies [6,14]. It was similarly found in studies involving diverse populations, like the United States [15], and Slovenian [8], Spanish [16], Turkish [17] and Danish [18] populations. In the studies from Dembinski et al. [15], Kastelic et al. [8], Navarro-López et al. [16] and Cabrejas-Olalla et al. [18], none of the individuals with self-reported intermediate eye color were classified as intermediate, identical to our results. Moreover, most intermediate cases of Cabrejas Ollala et al. [18] were misclassified as blue (121 out of 136), closely resembling our findings.

A relevant study by Andersen et al. [19] emphasizes that the misclassification of eye color represents a major challenge. The subjective perception of eye color and, consequently, self-assessment, may be a key issue. The authors propose an objective and continuous color measurement method and examine eye color using the custom designed software DIAT (digital iris analysis tool), which captures the brown and blue pixels of the iris [19]. However, their results showed a very high correlation between subjective assessment and the DIAT measurements, suggesting that subjective perception is not the cause of poor classification performance and is likely sufficient.

Other studies used different evaluation approaches to address the problem of assigning intermediate eye colors as well. The study by Meyer et al. [20] aimed at bypassing the intermediate eye color entirely by using a binary system. Thirty Danish participants classified 442 eye photographs using a three-way system consisting of blue, brown, and intermediate, and a two-way system lacking the intermediate category. Their results indicated that subjective perception aligns better with the two-category classification system, with only 38% agreement of eye colors in the three-category model and 79% agreement in the two-category model. Notably, none of the matches in the three-category model involved intermediate eye color.

One of the main problems is the classification of eye color into three distinct categories, as eye color is inherently a continuous phenotype. This reduces the statistical power considerably. Similar reasoning applies to hair and skin color. Paparazzo et al. [9] examined 238 southern Italian individuals using high-resolution images. They introduced a new quantitative and objective technique with clustering algorithms to better capture the continuous nature and fluid transition between eye colors. They compared their results with visual inspections of eye colors. Clustering improved accuracy compared to visual inspections only for brown eyes. In contrast, the results for blue eyes worsened after applying clustering and for intermediate eyes, accuracy remained at 0% both before and after clustering.

Behrens et al. [21] recently explored additional SNPs for eye color prediction and introduced the first population-specific online prediction tool for the admixed South Brazilian population, focusing on the differentiation of intermediate eye colors.

### 4.2. Hair Color

For hair color, black and blond could be estimated with high precision in our population study (accuracy of 95% and 68%, respectively; note that only eight individuals with black hair were included, limiting the accuracy of our results). However, discriminating between blond and brown hair was challenging. Eleven out of 73 blond participants were misclassified as brown and 32 out of 67 brown participants were misclassified as blond. The difficulty in distinguishing between blond and dark-haired individuals, as also observed in our study, has been reported previously in other publications [10,22,23]. The cause appears to be age-related darkening of hair color, which cannot be detected by the HIrisPlex-S system due to the lack of causal biomarkers for this process. Therefore, individuals who were blond in childhood and became brown in adulthood are more often misclassified [23]. In addition, the lower accuracy of hair color predictability may be caused by the difficulty in distinguishing between dark blond and light brown and the subjectivity of hair color self-assessment.

Navarro-López et al. [16] reported generally very low AUC values for all hair colors in their Spanish population, highlighting as a main issue the “high proportion of blond (54.05%) and black-haired (51.55%) individuals being misclassified as brown-haired”. In contrast, in our dataset, only 11 out of 73 (15%) blond-haired individuals were misclassified as brown. Similarly, Sari et al. [17] found a high misclassification rate of 41% (11/27) for self-reported blond individuals as brown in the Turkish population, indicating that blond hair prediction performed relatively worse in their cohort. This difference may partly be explained by the lower proportion of blonds in their study (18%) compared to ours (49%).

Concerning black-haired individuals, in our study, only one out of eight was correctly predicted as black, while most were misclassified as brown (6/8), resulting in a very low sensitivity of only 13%. In the Turkish study, in contrast, almost all self-reported black-haired individuals were correctly predicted as black by HIrisPlex-S (20/21), with only one misclassified as brown, resulting in a respective sensitivity of 96% [17]. Sari et al. [17] detected a similar prediction performance for brown-haired individuals. Only three out of 97 brown-haired individuals were misclassified (two as black and one as blond), leading to a sensitivity of 97% [17]. In our study, 32 out of 67 brown-haired individuals were misclassified as blond, resulting in a sensitivity of 52%. Results largely consistent with our findings regarding hair color distribution and prediction performance were achieved in the cohort of Salvo et al. [24] from 2022 including 540 Norwegian individuals (287 blond, 219 brown, 19 red, and 15 black). A total of 44% of brown-haired individuals were predicted as blond and 21% of blonds were predicted as brown. The authors suggested that age-dependent hair color changes and darkening in adulthood may explain these findings and contribute to the challenges in accurately classifying brown hair. Sari et al. [17] suggested that the higher accuracy of brown hair prediction in their population may be due to the fact that most brown-haired individuals were not affected by age-related color changes. In contrast, blond phenotypes are more common in Northern Europe and more often affected by age-related changes, contributing to higher rates of misclassification of brown hair as blond.

### 4.3. Skin Color

For skin color, in our population study, very pale skin was frequently misclassified as pale (5/5) and pale as intermediate (16/65), whereas very pale and intermediate could be easily differentiated from one another. In Walsh et al. [4] and Chaitanya et al. [5], it is noted that dark and light skin types are readily distinguishable from one another, whereas discrimination between lighter types, such as very pale, pale and intermediate, is more difficult. Their study population comprised 2025 individuals from 31 populations across Africa, Europe, East Asia, South Asia, the Americas, and Oceania. Some of them, however, were also included in “marker ascertainment, model building, or testing” [4]. By contrast, our sample is smaller (*n* = 152) and less diverse, restricted to northern Germany, and does not include individuals with dark or dark-to-black skin tones. Overall, the proportions of very pale, pale and intermediate skin colors are comparable between the two datasets. With respect to prediction accuracy, the AUC values are similar across the two studies for the intermediate category. For the very pale category, our performance is slightly better (86% vs. 74%), whereas for the pale category our performance is worse (66% vs. 72%). A key methodological difference lies in the phenotyping approach. Unlike our study, where participants self-reported their Fitzpatrick skin type, Walsh et al. [4] employed a trained dermatologist for classification. This method minimizes potential bias from self-assessment and reduces inter-observer variability in human color perception, since all assignments were made by a single expert. Both studies condensed Fitzpatrick categories III and IV into the intermediate category. Additionally, Walsh et al. [4] established a three-category classification system: light (Fitzpatrick I–IV), dark (Fitzpatrick V), and Dark-Black (Fitzpatrick VI). This simplified categorization yielded better prediction performance compared to the five-category model, with AUCs of 97% (light), 83% (dark), and 96% (Dark-Black). In addition, they evaluated an independent sample set (*n* = 194, including 17 different populations from Europe, the Middle East, Africa, and Asia). Therefore, to compare the results, it is better to focus on this smaller sample. However, they only considered the three-category system with AUCs of 92% for light, 74% for dark, and 94% for Dark-Black. Thus, collapsing all lighter categories (very pale, pale, and intermediate) into a single light group improves prediction accuracy and may be suitable for analyzing a diverse population but not a homogenous one like our northern German population. This highlights the system’s current limitation: it performs well in broadly distinguishing light from dark skin types but struggles to reliably separate finer gradations within the light spectrum.

The widely used Fitzpatrick scale is useful for categorizing skin color; however, assigning individuals to categories often relies on subjective rather than objective assessment. Yu et al. [25] proposed the use of a K-means clustering algorithm as a more objective alternative, since it yields reproducible and quantifiable outcomes. Similarly, Harvey et al. [26] emphasized the limitations of the Fitzpatrick scale and highlighted more objective approaches, such as UV light photography, which allows the direct assessment of epidermal melanin. While these methods may be advantageous in clinical research, they are not practical in forensic applications, where investigators often rely on subjective witness statements or DNA samples without access to the individual phenotype. Nevertheless, adopting a more objective classification system could enhance the accuracy of skin color prediction with HIrisPlex-S.

### 4.4. HIrisPlex-S Markers

In our study population, we found four SNPs to be monomorphic, meaning they were identical in all 152 individuals: rs312262906 (N29insA), rs1805006, rs201326893 (Y152OCH), and rs6119471. Three of these SNPs (rs312262906, rs1805006, and rs201326893) are variants of the *MC1R* gene, all of which were already described as quite rare by Walsh et al. [10]. This rarity may explain why they are monomorphic in our study population, as we only had four of the 152 individuals (3%) with red hair, and, even among these, the variant alleles of rs312262906 and rs201326893 are, as the study of Walsh et al. [10] noted, rare. Nevertheless, they remain important to include in the HIrisPlex-S system, because if a person is homozygous or compound heterozygous for these variants, the prediction probability of having pure red hair is 100%. Moreover, *MC1R* rs1805006 is important not only for hair color [10] but also for skin color [5], and *ASIP* rs6119471 is known to be relevant for eye color prediction [27] as well as skin color [5], particularly for discriminating non-light skin colors. Our northern German population, which only had very pale to intermediate skin, lacks these darker skin types. Thus, the homozygous status of the ancestral allele of this marker in our sample was not surprising.

### 4.5. Probability Threshold

The standard procedure for pigmentation category prediction is to select the category with the highest probability, as given by HIrisPlex-S. A probability threshold of 0.7 for eye and hair color was first mentioned in Walsh et al. [28], with the intention to increase the prediction accuracy. One drawback of the method was that, in our study, 74% of participants (110 out of 148) could not be classified into a hair color category, and 11% (16 out of 152) could not be classified into an eye color category when applying this threshold. The other disadvantage was the lack of improvement in accuracy in the remaining predictions. Paparazzo et al. [9] tested two thresholds (0.5 and 0.7) versus no thresholds using both visual classification and the clustering method. In conclusion, the new clustering technique presented by Paparazzo et al. [9] improved the classification of brown eyes but led to more frequent misclassification of blue eyes as intermediate.

### 4.6. Limitations

There are certain limitations associated with our study. First, the sample size was relatively small, and we only had four red-haired participants. In addition, data was collected using self-reported questionnaires, so all participant information was subjective. While this approach is not ideal, it reflects similar challenges encountered with witness statements in forensic practice, which are likewise subjective and not quantitatively measurable. Nevertheless, as suggested in other studies, an additional layer of objectivity could have been introduced, for example, by involving independent raters (e.g., a dermatologist [4,5] or rating of a professional examiner in comparison to standard images [29]). However, such an approach would have required considerable additional effort and money.

Another limitation is the absence of standardized scales for hair and eye color. To improve classification accuracy, the use of standardized scales for hair and eye color (e.g., clustering, PIE score [19] or the Kolberg Iris Color Chart from Valenzuela et al. [30]) could be beneficial. For skin color, the Fitzpatrick scale was applied by the participants themselves, while in other studies, more objective measurements like spectrophotometry, chemical examination of the two types of melanin [30] or computational evaluation with custom-made software with self-defined scores were used [19]. The challenge here may lie less in the classification system itself and more in the nature of individual human color perception (Bosten [31]; Neitz & Neitz [32]). Bortolotti et al. [33] highlight that color perception is influenced by a combination of genetic, physiological, developmental, environmental and sociocultural factors, including language. These aspects may partly explain the challenges in reliably distinguishing between dark-blond and light-brown hair, or in correctly assigning intermediate eye color categories. A further constraint concerns age-related changes in hair color [22,23]. To enhance predictive performance, further research on genes and SNPs associated with color change is needed. Otherwise, hair color in individuals affected by aging processes will continue to be predicted as lighter than their actual shade.

### 4.7. Practical Applications

Our study highlights both the potential and the limitations of pigmentation phenotype prediction. While such predictions can provide useful information for identification and generate investigative leads, it must be considered that the outputs of the HIrisPlex-S system are probabilistic and may vary substantially in their accuracy. While prediction accuracy is high for some categories, it is considerably lower for others, particularly for intermediate phenotypes. The results should therefore not be interpreted as definitive, but rather as probabilistic indications. Importantly, their use should not lead to an undue narrowing of investigations toward specific population groups, as this carries the risk of reinforcing biases or contributing to discriminatory practices. A responsible and ethically informed application of FDP is essential and requires appropriate training and clear guidelines for practitioners [34,35].

## 5. Conclusions

The significance of this study lies not in methodological innovations—since we used established markers and well-known methods—but in its role as the first population-based study on this topic in a German cohort. The high prevalence of intermediate phenotypes (31% intermediate eye color) highlights the limitations of current pigmentation trait classifications and demonstrates the need for more nuanced interpretations. The study emphasizes the necessity of novel approaches to enhance predictive precision, including further research on the genes responsible for hair color alterations due to the aging process and intermediate eye colors. Moreover, it raises awareness of the appropriate use of the HIrisPlex-S tool, which is more suitable for predicting broad phenotype categories, like light (e.g., blond hair color, blue eye color, and pale skin color) vs. dark (e.g., black hair color, brown eye color and intermediate skin color) rather than specific color nuances (e.g., light-brown or dark-blond hair color, and intermediate eye color). Given its current state of development, the HIrisPlex-S tool should be used with the utmost caution in police investigations, and a careful interpretation—and thus an assessment of the strength of the phenotype results by forensic experts—is absolutely essential.

## Figures and Tables

**Table 1:** Frequencies of self-reported hair, eye and skin color. The most frequent phenotype for each trait is highlighted in bold.

Phenotype	Color	Absolute Frequency (Percentage)
Eye color	blue brown intermediate	**67 (44.08%)** 38 (25.00%) 47 (30.92%)
Hair color ^1^	blond brown black red ^1^	**73 (48.03%)** 67 (44.08%) 8 (5.26%) 4 (2.63%)
Skin color	very pale pale intermediate dark dark-to-black	5 (3.29%) **65 (42.76%)** 82 (53.95%) 0 (0.00%) 0 (0.00%)

^1^ The four individuals with red hair were excluded from the performance analysis due to small sample size.

**Table 2:** HIrisPlex-S predictions of eye (A), hair (B) and skin color (C).

**(A) Eye color**						
**HIrisPlex-S result**	**Self-reported**					
	**Blue**		**Brown**	**Intermediate**		**Absolute frequency (%)**
**Blue**	66		2	34		102 (67.11%)
**Brown**	1		36	13		50 (32.90%)
**Intermediate**	0		0	0		0 (0.00%)
**Absolute frequency (%)**	67 (44.08%)		38 (25.00%)	47 (30.92%)		152 (100.00%)
**(B) Hair color**						
**HIrisPlex-S result**	**Self-reported**					
	**Blond**		**Brown**	**Black**		**Absolute frequency (%)**
**Blond**	59		32	1		92 (62.16%)
**Brown**	11		35	6		52 (35.14%)
**Black**	0		0	1		1 (0.68%)
**Red**	3		0	0		3 (2.03%)
**Absolute frequency (%)**	73 (49.32%)		67 (45.27%)	8 (5.41%)		148 (100.00%)
**(C) Skin color**						
**HIrisPlex-S result**	**Self-reported**					
	**Very pale**	**Pale**	**Intermediate**	**Dark**	**Dark-to-black**	**Absolute frequency (%)**
**Very pale**	0	1	0	0	0	1 (0.07%)
**Pale**	5	48	43	0	0	96 (55.92%)
**Intermediate**	0	16	37	0	0	53 (34.87%)
**Dark**	0	0	2	0	0	2 (1.32%)
**Dark-to-black**	0	0	0	0	0	0 (0.00%)
**Absolute frequency (%**)	5 (3.29%)	65 (42.76%)	82 (53.95%)	0 (0.00%)	0 (0.00%)	152 (100.00%)

For HIrisPlex-S, the predicted category was defined as the one with the highest predicted probability.

**Table 3:** Diagnostic performance measures of HIrisPlex-S for eye, hair and skin color.

Diagnostic Performance Measures	Phenotype											
	Eye Color			Hair Color				Skin Color				
	Blue	Intermediate	Brown	Blond	Brown	Black	Red	Very Pale	Pale	Intermediate	Dark	Dark-to-Black
**AUC (%)** **(95% CI)**	88.58 (83.38–93.78)	61.36 (52.25–70.47)	95.63 (92.73–98.52)	71.15 (62.82–79.48)	67.85 (59.02–76.67)	82.72 (68.63–96.82)	NA ^1^	86.26 (74.63–97.89)	65.94 (57.31–74.57)	72.06 (63.88–80.23)	NA ^2^	NA ^2^
**Sensitivity (%)** **(95% CI)**	98.51 (90.86–99.92)	0.00 (0.00–9.41)	94.74 (80.93–99.08)	80.82 (69.58–88.75)	52.24 (39.77–64.45)	12.5 (0.66–53.32)	NA ^1^	0.00 (0.00–53.71)	73.85 (61.23–83.61)	45.12 (34.24–56.46)	NA ^2^	NA ^2^
**Specificity (%)** **(95% CI)**	57.65 (46.46–68.14)	100.00 (95.60–100.00)	87.72 (79.93–92.88)	56.00 (44.10–67.29)	79.01 (68.26–86.96)	100.00 (96.67–100.00)	97.97 (93.73–99.48)	99.32 (95.70–99.96)	44.83 (34.28–55.84)	77.14 (65.28–85.99)	98.68 (94.84–99.77)	100.00 (96.93–100.00)
**PPV (%)** **(95% CI)**	64.71 (54.55–73.74)	NA ^3^	72.00 (57.29–83.33)	64.13 (53.39–73.67)	67.31 (52.78–79.28)	100 (5.46–100)	0 (0–69.00)	0 (0.00–94.54)	50.00 (40.19–59.81)	69.81 (55.49–81.26)	0 (0–80.21)	NA ^4^
**NPV (%)** **(95% CI)**	98.00 (87.99–99.90)	69.08 (61.00–76.18)	98.04 (92.41–99.66)	75.00 (61.35–85.20)	66.67 (56.22–75.76)	95.24 (90.06–97.90)	100 (96.78–100)	96.69 (92.04–98.78)	69.64 (55.74–80.84)	54.55 (44.26–64.48)	100 (96.89–100)	100 (96.93–100)
**Prevalence (%)** **(95% CI)**	44.08 (36.11–52.35)	30.92 (23.82–39.00)	25.00 (18.50–32.79)	49.32 (41.06–57.62)	45.27 (37.15–53.64)	5.41 (2.54–10.73)	NA ^1^	3.29 (1.22–7.91)	42.76 (34.86–51.04)	53.95 (45.70–61.99)	0 (0–3.07)	0 (0–3.07)
**Accuracy (%)** **(95% CI)**	75.66 (67.91–82.08)	69.08 (61.00–76.18)	89.47 (83.20–93.67)	68.24 (60.01–75.51)	66.89 (58.62–74.27)	95.27 (90.13–97.91)	97.97 (93.73–99.48)	96.05 (91.23–98.39)	57.24 (48.96–65.14)	59.87 (51.60–67.64)	98.68 (94.84–99.77)	100 (96.93–100)

AUC: area under the curve, CI: confidence interval, PPV: positive predictive value, NPV: negative predictive value, NA: not available. ^1^ Not available because persons with red hair were excluded from the analysis due to small sample size, ^2^ not available because no person had dark or dark-to-black skin color, ^3^ not available because no person was classified as having intermediate eye color, ^4^ not available because no person was classified as having very pale or dark-to-black skin color. For HIrisPlex-S, the predicted category was defined as the one with the highest predicted probability.

## Data Availability

The data presented in this study is available on request from the corresponding author due to ethical reasons (privacy).

## Supplementary Materials

The following supporting information can be downloaded at: https://www.mdpi.com/article/10.3390/genes17040452/s1, Table S1: Recoding of questionnaire categories into HIrisPlex-S categories, Table S2: Characterization of skin color categories for the questionnaire, Table S3: Single base extension primer of the 24-plex assay with corresponding mutation pattern and amplicon length, Table S4: Single base extension primer of the 17-plex assay with corresponding mutation pattern and amplicon length, Table S5: 41 SNP profiles of our population (*n*=152) in comparison with a European Subgroup from NCBI ALFA, Table S6: HIrisPlex-S predictions of eye and hair color with probability threshold of 0.7, Table S7: Diagnostic performance measures of HIrisPlex-S for eye and hair color with probability threshold, Figure S1: Original questionnaire German, Figure S2: Questionnaire English, Figure S3: Example electropherogram of the HIrisPlex-S 24-Plex, Figure S4: Example electropherogram of the HIrisPlex-S 17-Plex, Figure S5: Boxplots and ROC curves for the probability of eye colors as given by HIrisPlex-S, Figure S6: Boxplots and ROC curves for the probability of hair colors as given by HIrisPlex-S.
