# Supplementary material for: Evaluation of the Prediction Potential of the HIrisPlex-S System in a North German Population
Source: Genes (Basel). 2026 Apr 13;17(4):452. doi: 10.3390/genes17040452 (PMC13116637; doi:10.3390/genes17040452)
Supplement: Supplementary file 1 [file genes-17-00452-s001.zip › genes-4226399-supplementary.pdf]

Supplementary Tables

Supplementary Table S1: Recoding of questionnaire categories into HIrisPlex-S categories, *others/individual - “Others” refers to responses from participants that did not directly correspond to the four predefined categories. These were “individually” classified into the categories by the study team.

Phenotype	Self-reported category	HIrisPlex-S category
**Hair color**	blond<br>red<br>brown<br>black<br>others*	blond<br>red<br>brown<br>black<br>individual*
**Eye color**	blue<br>blue-grey	blue
	brown<br>brown-grey<br>brown-hazel<br>dark brown<br>inner circle brown, outer circle more yellow<br>light brown<br>nut brown	brown
	blue-brown<br>blue-green<br>blue-grey-green<br>blue-light brown<br>green<br>green-blue<br>green-brown-grey<br>green-brown<br>green-grey<br>grey<br>brown-green<br>brown-grey-green<br>brown (light green)<br>olive green-brown	intermediate
**Skin color**	1	very pale
	2	pale
	3<br>4	intermediate
	5	dark
	6	dark to black

Supplementary Table S2: Characterization of skin color categories for the questionnaire

HIrisPlex-S-Tool skin color category	Self-reported skin color		
**Skin color**	**Number**	**Natural skin color**	**Tanning after sunbathing**
**Very pale**	1	very pale, very often freckled	never tans
**Pale**	2	pale, often freckled	tans minimally
**Intermediate**	3	light brown	usually tans
	4	moderate brown	tans well
**Dark**	5	dark brown	rarely burns
**Dark-to-black**	6	dark brown to black	never burns

Supplementary Table S3: Single base extension primer of the 24-plex assay with corresponding mutation pattern and amplicon length

**HIrisPlex-S 24-Plex**				
SNP	SBE-Primer (5'-3')	Alleles	Amplicon [bp]	Conc [µm]
N29insA	CCAGCTGGGGCTGGCTGCCAA	C/A	21	3.9
rs1805005	GGTGGAGAACGCGCTGGTG*	G/T	19	2
rs1805006	ttttttttttttttttttttCTGCCTGGCCTTGTCGGA	C/A	38	2.4
rs2228479	ttttttttttttttttttttttttttCTGGTGAGCGGGAGCAAC	G/A	44	1.5
rs11547464	ttttttttttttGCCATCGCCGTGGACC	G/A	28	0.3
rs885479	ttttttttttttttttttGATGGCCGCAACGGCT*	C/T	34	4
rs1805008	ttttttttACAGCATCGTGACCCTGCCG*	C/T	28	0.38
Y152OCH	ttttttttttttttttttttttttttttttCATCTTCTACGCACTGCGCTA	C/A	51	0.4
rs1805007	tttttttttttttttttttttttttCTCCATCTTCTACGCACTG	C/T	44	1.1
rs1110400	ttttttttttttttttttttttttttttttCTTCTACGCACTGCGCTACCACAGCA	T/C	56	0.2
rs1805009	ttttttttttttttttttttttttttttttATCTGCAATGCCATCATC	G/C	48	0.36
rs28777	tttttttttttttttttttttttttttttttttttttttCATGTGATCCTCACAGCAG	C/A	58	1.2
rs16891982	ttttttttttttttttttttttttttttttttttttttttttttAAACACGGAGTTGATGCA	C/G	62	1.8
rs12821256	tttttttGGAGCCAAGGGCATGTTACTACGGCAC*	A/G	34	0.12
rs4959270	ttttttttttttttttttttttttttttttttttttttttttttttGGAACACATCCAAACTATGACACTATG*	C/A	73	0.1
rs12203592	tttttttttttttttttttttttttttttttttttttttttttttTCCACTTTGGTGGGTAAAAGAAGG	C/T	69	0.2
rs1042602	ttttttttttttttttttttttttttttttttttttttttttttttttttttTCAATGTCTCTCCAGATTTCA	G/T	73	1.25
rs1800407	tttttttttttttttttttttttttttttttttttttttttttttttttttttttttttttGCATACCGGCTCTCCC	G/A	77	0.3
rs2402130	ttttttttttttttttttttttttttttttttttttttttttttttttttttttttttttTGAACCATACGGAGCCCGTG	G/A	80	0.75
rs12913832	tttttttttttttttttttttttttttttttttttttttttttttttTAGCGTGCAGAACTTGACA*	T/C	66	0.7
rs2378249	tttttttttttttttttttttttttttttttttttttttttttttttttttttttttCCACACCTCTCCTCAGCCCA*	C/T	77	0.05
rs12896399	tttttttttttttttTCTTTAGGTCAGTATATTTTGGG*	G/T	38	1.0
rs1393350	tttttttttttttttttttttttttttttttttttttttttttttttttttttttCATTTGTAAAAGACCACACAGATTT*	C/T	80	2.2
rs683	ttttttttttttttttttttttGCTTTGAAAAGTATGCCTAGAACTTTAAT*	G/T	51	0.6

* Total length of the SBE primers was changed compared to the publication (6), SBE: single base extension, bp: base pair, Conc.: SBE input concentration per sample in µm

Supplementary Table S4: Single base extension primer of the 17-plex assay with corresponding mutation pattern and amplicon length

**HIrisPlex-S 17-Plex**				
SNP	SBE-Primer (5'-3')	Alleles	Amplicon [bp]	Conc [µm]
rs3114908	ttttttttttAGAGAAGGGTCAAGCACTT	T/C	29	0.1
rs1800414	tttttttttttCAGAATCCCGTCAGATATCCTA	T/C	33	0.3
rs10756819	ttttttttttttGGACCAGTTATTTTGGGTTTGGA	G/A	35	2.5
rs2238289	ttttttttttttttttttttGAGATTGGAAGATTGGAGCC	T/C	40	0.5
rs17128291	tttttttttttttttttttttCAATGTGCACTGGATTAAAAGTC	T/C	44	1.0
rs6497292	ttttttttttttttttttttttttttGTCTCCTGTGTCTTCATCCT	T/C	46	0.2
rs1129038	ttttttttttttttttttttttttttttttttttttGAGCCAGGCAGCAGAGC	G/A	53	0.4
rs1667394	tttttttttttttttttttttttttttttttttttGCAGCAATTCAAAACGTGCATA*	C/T	57	0.2
rs1126809	ttttttttGTATTTTTGAGCAGTGGCTCC*	G/A	29	0.05
rs1470608	ttttttttttttttttttttttttttttttttCATTCTCTCTTAAAAATATTAATTTGCACC	C/A	62	2.0
rs1426654	tttttttttttttttttttttttttGTCTCAGGATGTTGCAGGC*	A/G	44	0.8
rs6119471	ttttttttttttttttttttttttttttttttttttttttttttttGAAGGAAGAGTGAAAATGCGTAA	G/C	69	0.5
rs1545397	tttttttttttttttttttttttttttttttttttttttttGTACAACTTTGTGAATATACTAAAATAC*	A/T	69	1.5
rs6059655	tttttttttttttttttttttttttttttttttACGAGCTGATGCCCTGAGCA**	T/C	53	0.3
rs12441727	ttttttttttttttttttttttttGGCTCAGTGTGGCCTT*	G/A	40	0.5
rs3212355	ttttttttttttttttttttttttttttttttttttttttttttttttttCCGAAGCCCAGCAGG*	G/A	65	0.75
rs8051733	tttttttttttttttttttttttttttttttttttttttttttttttttCACCCTGCCTGTCTCG*	T/C	65	2.5

* Total length of the SBE primers was changed compared to the publication; **specific sequence increased by four Nucleotides (5), SBE: single base extension, bp base pair, Conc.: SBE input concentration per sample in µm

Supplementary Table S5: 41 SNP profiles of our population (n=152) in comparison with a European Subgroup from NCBI ALFA

Gene	SNP	Minor Allel HIrisPlex (ALT)	Major Allel HIrisPlex (REF)	Hom. Major allele	Het.	Hom. Minor allele	MAF (%) This study	MAF (%) NCBI ALFA
MC1R	rs312262906	A	C	152	0	0	0.00	0.32
MC1R	rs11547464	A	G	150	2	0	0.66	0.80
MC1R	rs885479	T	C	135	17	0	5.59	4.47
MC1R	rs1805008	T	C	126	25	1	8.88	7.49
MC1R	rs1805005	T	G	106	45	1	15.46	12.76
MC1R	rs1805006	A	C	152	0	0	0.00	0.98
MC1R	rs1805007	T	C	137	14	1	5.26	7.24
MC1R	rs1805009	C	G	151	1	0	0.33	1.51
MC1R	rs201326893	A	C	152	0	0	0.00	0.07
MC1R	rs2228479	A	G	125	25	2	9.54	8.63
MC1R	rs1110400	C	T	147	5	0	1.65	0.95
SLC45A2	rs28777	C	A	144	8	0	2.63	3.20
SLC45A2	rs16891982	C	G	143	9	0	2.96	4.49
KITLG	rs12821256	G	A	118	32	2	11.84	10.54
EXOC2	rs4959270	A	C	44	77	31	45.72	47.88
IRF4	rs12203592	T	C	130	21	1	7.57	15.50
TYR	rs1042602	T	G	61	71	20	36.51	37.41
OCA2	rs1800407	A	G	138	14	0	0.33	7.20
SLC24A4	rs2402130	G	A	102	45	5	18.09	19.90
HERC2	rs12913832	T	C	101	46	5	18.42	25.86
PIGU/ASIP	rs2378249	C	T	110	38	4	15.13	15.60
SLC24A4	rs12896399	T	G	41	83	28	45.72	43.82
TYR	rs1393350	T	C	94	48	10	22.37	26.17
TYRP1	rs683	G	T	58	73	21	37.83	35.11
ANKRD11	rs3114908	T	C	65	74	13	32.90	32.60
OCA2	rs1800414	C	T	151	1	0	0.33	0.06
BNC2	rs10756819	G	A	60	77	15	35.20	33.80
HERC2	rs2238289	C	T	123	27	2	10.20	11.70
SLC24A4	rs17128291	C	T	110	34	8	16.45	15.90
HERC2	rs6497292	C	T	137	15	0	4.93	6.35
HERC2	rs1129038	G	A	100	47	5	18.75	26.38
HERC2	rs1667394	C	T	115	34	3	13.16	17.42
TYR	rs1126809	A	G	87	54	11	25.00	27.48
OCA2	rs1470608	A	C	105	39	8	18.09	14.95
SLC24A5	rs1426654	G	A	150	2	0	0.66	0.36
ASIP	rs6119471	C	G	152	0	0	0.00	0.12
OCA2	rs1545397	T	A	129	21	2	8.22	6.85
RALY	rs6059655	T	C	133	17	2	6.91	7.68
OCA2	rs12441727	A	G	112	33	7	15.26	10.38
MC1R	rs3212355	A	G	151	1	0	0.33	0.01
DEF8	rs8051733	C	T	76	65	11	28.62	31.13

Hom. homozygote, Het. heterozygote, MAF minor allele frequency, NCBI National Center for Biotechnology Information, ALFA Allele Frequency Aggregator, MAF NCBI ALFA minor allele frequency from the NCBI database of the European subgroup of the ALFA project from the website https://www.ncbi.nlm.nih.gov/snp [cited 2025 Sept 25].

Supplementary Table S6: **HIrisPlex-S predictions of eye and hair color with probability threshold of 0.7**

A) Eye color

**HIrisPlex-S result (>0.7)**	**Self-reported**			
	**Blue**	**Brown**	**Intermediate**	**Absolute frequency (%)**
**Blue**	66	1	34	101 (74.27%)
**Brown**	1	28	6	35 (25.74%)
**Intermediate**	0	0	0	0 (0.00%)
**Absolute frequency (%)**	67 (49.27%)	29 (21.32%)	40 (29.41%)	136 (100.00%)

B) Hair color

**HIrisPlex-S Result (>0.7)**	**Self-reported**			
	**Blond**	**Brown**	**Black**	**Absolute frequency (%)**
**Blond**	18	9	0	27 (71.05%)
**Brown**	1	6	2	9 (23.68%)
**Black**	0	0	1	1 (2.63%)
**Red**	1	0	0	1 (2.63%)
**Absolute frequency (%)**	20 (52.63%)	15 (39.47%)	3 (7.90%)	38 (100.00%)

Only individuals with a predicted (eye or hair color) probability larger than 0.7 were included in this analysis. For HIrisPlex-S, the predicted category was defined as the one with the highest predicted probability.

Supplementary Table S7: Diagnostic performance measures of HIrisPlex-S for eye and hair color with probability threshold

Diagnostic performance measures	Phenotype						
	Eye color			Hair color			
	Blue	Intermediate	Brown	Blond	Brown	Black	Red
**AUC (%) (95% CI)**	86.28 (80.20-92.35)	60.66 (51.06-70.26)	97.00 (94.58-99.42)	79.31 (64.47-94.14)	60.43 (41.03-0.7984)	96.19 (87.92-100.00)	NA [1]
**Sensitivity (%) (95% CI)**	98.51 (90.86-99.92)	0.00 (0-10.91)	96.55 (80.37-99.82)	90.00 (66.87-98.25)	40.00 (17.46-67.11)	33.33 (1.77-87.47)	NA [1]
**Specifity (%) (95% CI)**	49.28 (37.15-61.48)	100.00 (95.21-100.00)	93.46 (86.52-97.10)	45.00 (23.83-67.95)	86.96 (65.33-96.57)	100.00 (87.69-100.00)	100.00 (88.29-100.00)
**PPV (%) (95% CI)**	65.35 (55.15–74.36)	NA [2]	80.00 (62.54-90.94)	66.67 (46.02-82.77)	66.67 (30.92-90.96)	100.00 (5.46-100.00)	0.00 (0.00-94.54)
**NPV (%) (95% CI)**	50.74 (42.07-59.36)	70.59 (62.07-77.93)	78.68 (70.66-85.04)	81.82 (47.76-96.79)	68.97 (49.05-84.02)	94.60 (80.47-99.06)	100.00 (88.29-100.00)
**Prevalence (%) (95% CI)**	49.27 (40.64-57.93)	29.41 (22.07-37.93)	21.32 (14.96-29.34)	52.63 (36.05-68.69)	39.47 (24.49-56.55)	7.90 (2.06-22.48)	0.00 (0.00-11.43)
**Accuracy (%) (95% CI)**	73.53 (65.15-80.55)	70.59 (62.07-77.93)	94.12 (88.36-97.24)	71.05 (53.89-84.02)	68.42 (51.21-81.96)	94.74 (80.93-99.08)	97.37 (84.57-99.86)

AUC area under the curve, CI confidence interval, PPV positive predictive value, NPV negative predictive value, NA not available, [1] not available because persons with red hair were excluded from the analysis due to low sample size, [2] not available because no person was classified as having intermediate eye color. For HIrisPlex-S, the predicted category was defined as the one with probability threshold.

Supplementary Figures

CHARITÉ
UNIVERSITÄTSMEDIZIN BERLIN

Institut für Rechtsmedizin und
Forensische Wissenschaften
Direktor: Prof. Dr. Michael Tsokos

Abteilung Forensische Genetik
Augustenburger Platz 1
13353 Berlin
Studienkoordination: Dr. Maria Seidel

Fragebogen

Geschlecht

DNA Nummer

☐ weiblich ☐ männlich ☐ divers

Einschätzung der Haarfarbe

☐ blond ☐ braun ☐ rot ☐ schwarz ☐ andere _______________

Farbton der Haare

☐ hell ☐ dunkel

Fand im Kindes- oder Jugendalter eine Verdunklung der Haarfarbe statt?

☐ ja ☐ nein

Augenfarbe: ____

Hautfarbe: ☐ ☐ ☐ ☐ ☐ ☐

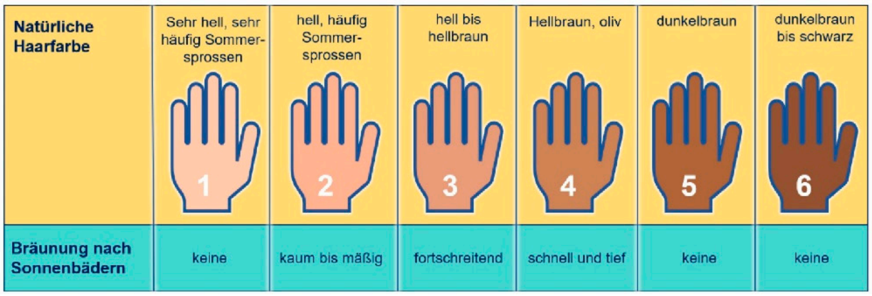

____

Datum

Version 2, 05.07.2021 Seite 1 von 1

Supplementary Figure S1: original questionnaire German

CHARITÉ UNIVERSITÄTSMEDIZIN BERLIN

Institut für Rechtsmedizin und
Forensische Wissenschaften
Direktor: Prof. Dr. Michael Tsokos

Abteilung Forensische Genetik
Augustenburger Platz 1
13353 Berlin
Studienkoordination: Dr. Maria Seidel

Questionnaire

DNA Number

Sex

☐ female ☐ male ☐ other

Hair color

☐ blond ☐ brown ☐ red ☐ black ☐ other ______________

Color shade of the hair

☐ light ☐ dark

Did the hair color darken during childhood or adolescence?

☐ yes ☐ no

Eye color: ____

Skin color: ☐ ☐ ☐ ☐ ☐ ☐

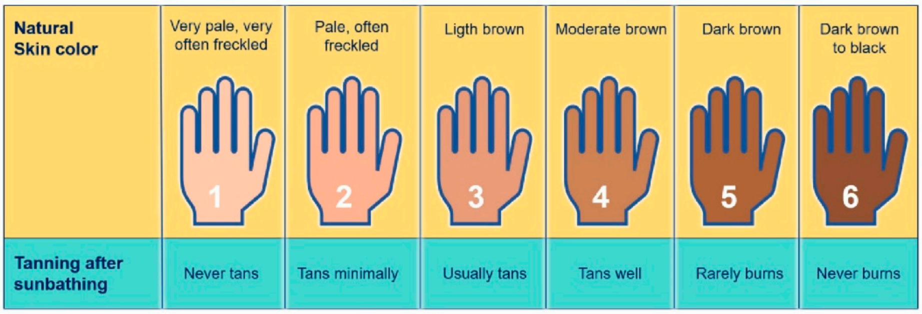

____
Date

Supplementary Figure S2: questionnaire English

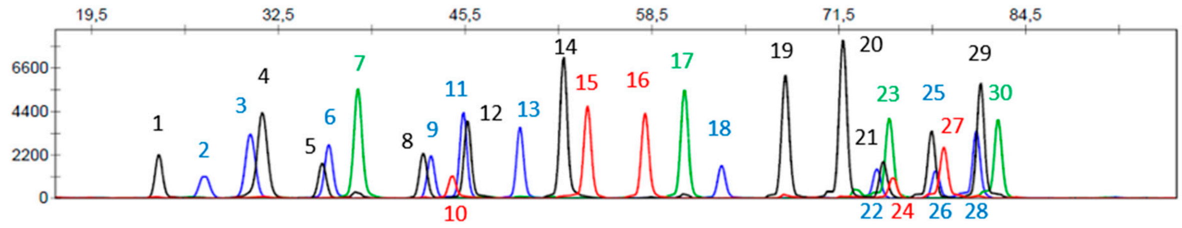

1 - N29insA_C
2 - rs1805005_G
3 - rs11547464_G
4 - rs1805008_C
5 - rs885479_C
6 - rs12821256_G
7 - rs12821256_A
8 - rs1805006_C
9 - rs12896399_G
10 - rs12896399_T
11 - rs2228479_G
12 - rs1805007_C
13 - rs1805009_G
14 - Y152OCH_C
15 - rs683_T
16 - rs111400_T
17 - rs28777_A
18 - rs16891982_G
19 - rs12913832_C
20 - rs12203592_C
21 - rs4959270_C
22 - rs1042602_G
23 - rs4959270_A
24 - rs1042602_T
25 - rs2378249_C
26 - rs1800407_G
27 - rs2378249_T
28 - rs2402130_G
29 - rs1393350_C
30 - rs2402130_A

Supplementary Figure S3: Example electropherogram of the HIrisPlex-S 24-Plex

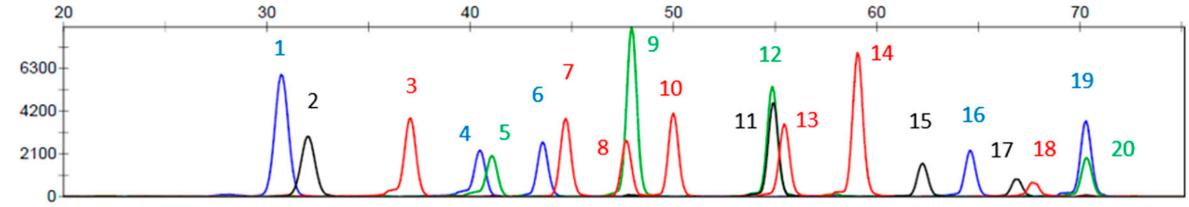

1 - rs1126809_G
2 - rs3114908_C
3 - rs1800414_T
4 - rs10756819_G
5 - rs10756819_A
6 - rs12441727_G
7- rs2238289_T
8 - rs17128291_T
9 - rs1426654_A
10 - rs6497292_T
11 - rs6059655_C
12 - rs1129038_A
13 - rs6059655_T
14 - rs1667394_T
15 - rs1470608_C
16 - rs3212355_G
17 - rs80551733_C
18 - rs8051733_T
19 - rs66119471_G
20 - rs1545397_A

Supplementary Figure S4: Example electropherogram of the HIrisPlex-S 17-Plex

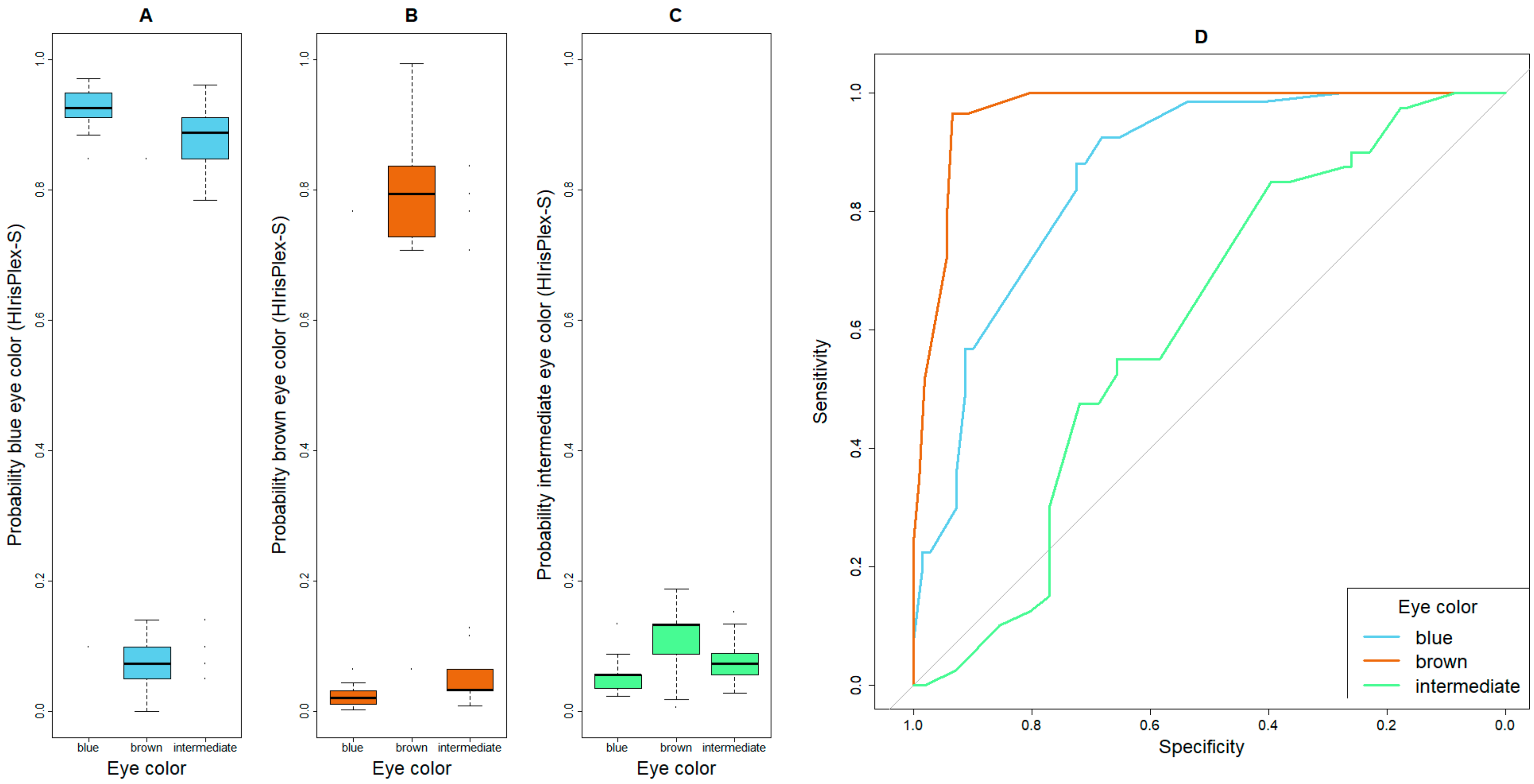

Supplementary Figure S5: Boxplots and ROC curves for the probability of eye colors as given by HIrisPlex-S.

A: Boxplot for the probability of blue eye color, B: Boxplot for the probability of brown eye color, C: Boxplot for the probability of intermediate eye color, D: ROC curves of blue, brown and intermediate eye color. Only individuals with a predicted eye color probability larger than 0.7 were included in this analysis.

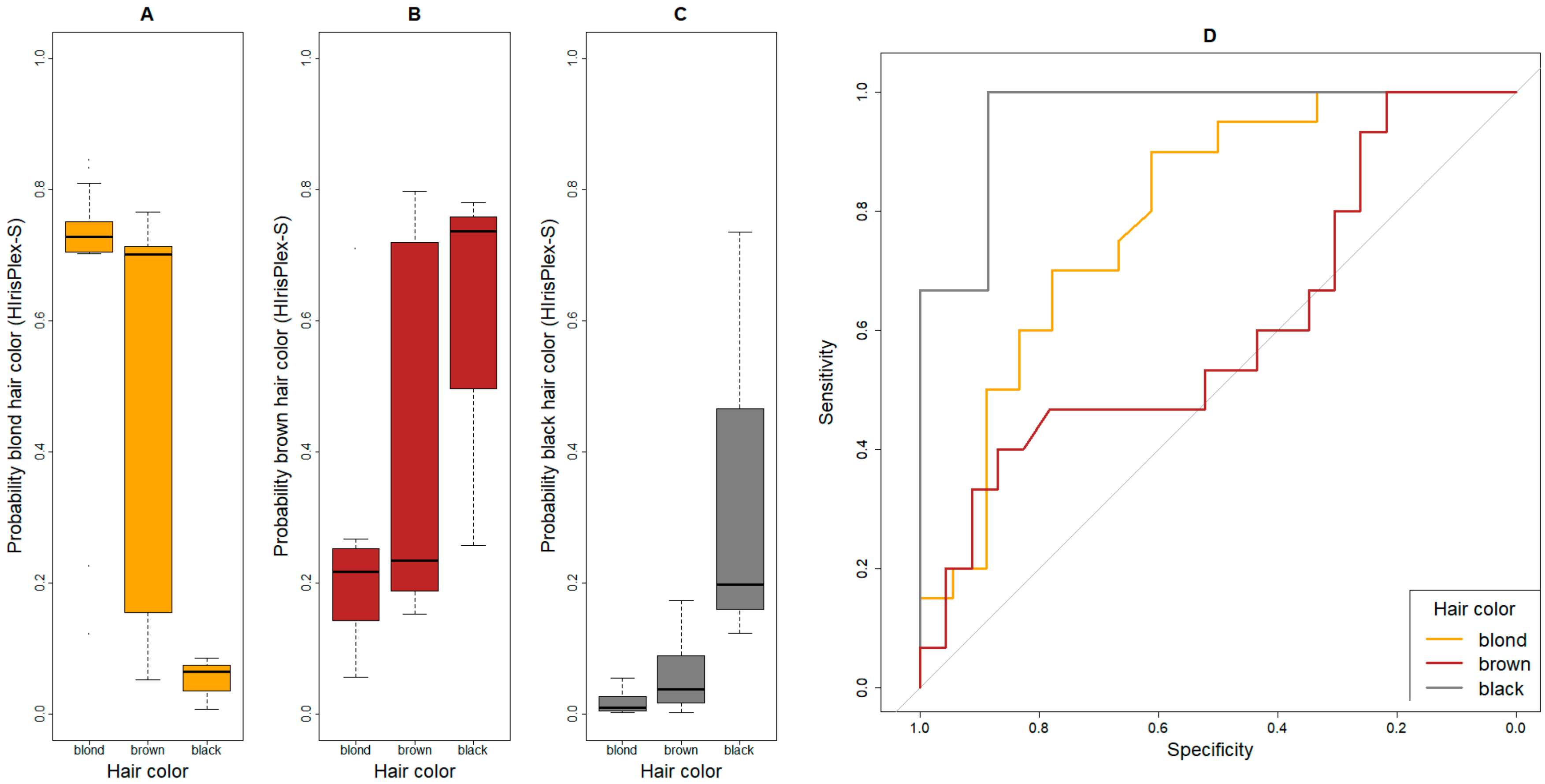

Supplementary Figure S6: Boxplots and ROC curves for the probability of hair colors as given by HIrisPlex-S.
A: Boxplot for the probability of blond hair color, B: Boxplot for the probability of brown hair color, C: Boxplot for the probability of black hair color, D: ROC curves of blond, brown and black hair color. Only individuals with a predicted hair color probability larger than 0.7 were included in this analysis.
